# Functional Analysis of the Ferric Uptake Regulator Gene *fur* in *Xanthomonas vesicatoria*

**DOI:** 10.1371/journal.pone.0149280

**Published:** 2016-02-24

**Authors:** Huiqin Liu, Chunling Dong, Tingchang Zhao, Jucai Han, Tieling Wang, Xiangzhen Wen, Qi Huang

**Affiliations:** 1 State Key Laboratory for Biology of Plant Diseases and Insect Pests, Institute of Plant Protection, Chinese Academy of Agricultural Sciences, Beijing, China; 2 College of Horticulture and Landscape, Tianjin Agricultural University, Tianjin, China; 3 Agronomy College, Shanxi Agricultural University, Taigu, Shanxi, China; 4 Horticulture College, Shanxi Agricultural University, Taigu, Shanxi, China; 5 Floral and Nursery Plants Research Unit, Agricultural Research Service, U. S. Department of Agriculture, Beltsville, Maryland, United States of America; East Carolina University School of Medicine, UNITED STATES

## Abstract

Iron is essential for the growth and survival of many organisms. Intracellular iron homeostasis must be maintained for cell survival and protection against iron toxicity. The ferric uptake regulator protein (Fur) regulates the high-affinity ferric uptake system in many bacteria. To investigate the function of the *fur* gene in *Xanthomonas vesicatoria* (*Xv*), we generated a *fur* mutant strain, fur-m, by site-directed mutagenesis. Whereas siderophore production increased in the *Xv fur* mutant, extracellular polysaccharide production, biofilm formation, swimming ability and quorum sensing signals were all significantly decreased. The *fur* mutant also had significantly reduced virulence in tomato leaves. The above-mentioned phenotypes significantly recovered when the *Xv fur* mutation allele was complemented with a wild-type *fur* gene. Thus, Fur either negatively or positively regulates multiple important physiological functions in *Xv*.

## Introduction

Iron is an essential trace metal element for living organisms because it serves as an enzymatic cofactor and a component of electron transport proteins. Phytopathogenic bacteria use iron as an environmental signal to regulate virulence genes [1]. However, iron can also be toxic when the intracellular concentration exceeds a critical level. This condition can induce detrimental oxidative stress by promoting Fe (II)-mediated forms of reactive oxygen via Fenton reactions [1,2]. Therefore, most bacteria maintain iron homeostasis via the ferric uptake regulator protein (Fur) [3]. The promoter regions of genes negatively regulated by the Fur protein typically have a conserved 19-bp sequence termed the “Fur box” [2,4,5]. During plant-microbe interactions, Fur plays a significant role in maintaining iron balance to reduce the toxicity of reactive oxygen [6]. In addition, Fur has been shown to regulate the expression of key virulence factors in pathogenic bacteria [4]. The Fur protein is a global regulator because it regulates genes related to iron uptake as well as the expression of many genes related to chemotaxis, the tricarboxylic acid cycle, glycolysis, oxidative stress, resistance redox and quorum sensing [2,3,6–8].

Bacterial spot of tomato (*Solanum lycopersicum* L.) and pepper (*Capsicum annuum*) is caused by the Gram-negative bacterium, *Xanthomonas vesicatoria* (*Xv*). Previous research has identified four phenotypic xanthomonad groups: group A (*X*. *euvesicatoria*), group B (*Xv*), group C (*X*. *perforans*) and group D (*X*. *gardneri*) [9]. Chen (2010) determined the phenotypic groups of more than 100 *Xv* strains collected in China [10]. Pathogenic *Xv* strains attack leaves, stems, fruits, and flowers. Leaf spots are initially small, but when spots are numerous, foliage turns yellow and eventually dies, leading to the defoliation of the plants. This disease has been reported in Europe, Asia, the Americas and other parts of the world [9]. The pepper pathogenic strain has been sequenced and used as a well-established model for studying bacterium-plant interactions [11], including the type III secretion system and several virulence genes [12,13]. However, the function of Fur in *Xv* has not previously been elucidated. In this communication, we investigated the role of *fur* in *Xv* 17, a wild-type *Xv* strain belonging to group B of xanthomonads that was isolated from tomato fruit in Xinjiang, China [10].

## Materials and Methods

### Media and bacterial growth conditions

All *Xv* strains were routinely cultured in solid yeast extract-dextrose-CaCO_3_ (YDC) medium [14] or liquid Luria-Bertani medium (LB) [15] at 28°C with continuous shaking (180 rpm). *Escherichia coli* strains were cultured in LB medium at 37°C with continuous shaking (180 rpm). *Erwinia carotovora* strains were cultured in LB medium at 28°C with continuous shaking (180 rpm). Antibiotics were added to appropriate media at the following concentrations: gentamicin (Gm) 50 μg·ml^-1^, rifampin (Rif) 50 μg·ml^-1^, kanamycin (Km) 50 μg·ml^-1^, chloramphenicol (Cm) 20 μg·ml^-1^ and ampicillin (Ap) 100 μg·ml^-1^, respectively. All bacterial strains and plasmids used in this study are listed in Table 1.

**Table 1:** Bacterial strains and plasmids used in this study.

Designation	Relevant characteristics^a^	Source or reference
**Strains**		
*Xanthomonas* v*esicatoria*		
17	Wild-type, Rif ^R^	This study
fur-m	*fur* mutant strain, containing truncated *fur* gene and Gm cassette, Rif ^R^, Gm^R^	This study
fur-c	*fur* complementation strain, fur-m containing pMLfur, Rif^R^, Km^R^, Gm^R^	This study
*Escherichia coli*		
DH5α	Competent cells, Φ80 *lacZ*	TaKaRa (China)
pRK600	Helper strain in triparental matings, Cm^R^	[18]
*Agrobacterium tumefaciens*		
NTL4	Quorum-sensing reporter strain, containing traG::*lacZ*, *tra* reporter, Gm^R^	[55]
*Erwinia carotovora* subsp. *carotovora*		
3	Indicator strain for quorum sensing	This study
**Plasmids**		
PMD18-T	T-vector, Ap^R^	TaKaRa (China)
pK18mob	Cloning and suicide vector with a *sacB* gene, Km^R^	[17]
pKfur	pK18mob containing a 1572-bp fragment containing *fur* and its 3’ and 5’ sequences, Km^R^	This study
pKfurGm	pK18 containing wild-type *fur* with its 209-bp replaced by Gm cassette, Km^R^, Gm^R^	This study
pML123	Complementation plasmid, Km ^R^, Gm^R^	[16]
pMLfur	pML123 containing a 618-bp fragment of *fur* and its promoter region, Km^R^, Gm^R^	This study

^a^Rif^R^, Ap^R^, Km^R^, Gm^R^ and Cm^R^ indicate resistance to rifampicin, ampicillin, kanamycin, gentamicin and chloramphenicol, respectively.

### Construction of the *fur* mutant

The *fur* gene in the wild-type strain *Xv*17 was inactivated by homologous integration as described by Windgassen et al. [16], using the suicide vector pK18mob [17]. Primers for PCR amplification were designed using the free online program Primer 5.0 (Table 2). Each reaction mixture contained 0.5 μl of DNA template, 6.25 μl of 2×PCR Mix (TaKaRa, Bao Biological Engineering (Dalian) Co., Ltd, Dalian, China) and 0.5 μl of each primer for a total reaction volume of 12.5 μl. The PCR conditions were 94°C for 3 min, 25 cycles of 94°C for 30 s, 60°C for 30 s and 72°C for 90 s, followed by 72°C for 5 min. The 1,572-bp fragment of *Xv*17 amplified by the fur1 and fur2 primers contained a 411-bp coding region of the *fur* gene, as well as the 633- and 528-bp upstream and downstream sequences of the gene (Table 2). After confirmation by sequencing, the fragment was digested by *EcoR*I and *Hind*III and cloned into pK18mob to create plasmid pKfur. pKfur was digested with *BamH*I and *Nde*I and a 209-bp fragment inside the 411-bp *fur* gene region was replaced with a *Gm* gene cassette (855 bp) to create plasmid pKfurGm (Table 1). pKfurGm was introduced from *E*. *coli* DH5 into *Xv*17 by triparental conjugation using pRK600 [18] as a helper plasmid. Transconjugants were screened on YDC supplemented with 10% sucrose and antibiotics (Rif and Gm) and confirmed by PCR using the fur1 and fur2 primers. To confirm the presence of the Gm cassette in the transconjugants, Southern blotting was performed with primers Gm1 and 2 using the marker BM5000 (Biomed, 5,000 bp, 3,000 bp, 2,000 bp, 1,000 bp, 750 bp, 500 bp, 250 bp, 100 bp) as the probe. The confirmed *fur* mutant strain, fur-m (Table 2), was used for subsequent studies.

**Table 2:** PCR primers used in this study.

Primers	Sequence (5’-3’, restriction enzyme sites are underlined	Product of PCR
fur1	GAATTCATCGGTCCTGGGAGTC *EcoR*I	1572 bp
fur2	AAGCTTCGGCGTGGAAGTGA *Hind*III	
Gm1	GGATCCGACGCACACCGTGGAAA *BamH*I	855 bp
Gm2	CATATGGCGGCGTTGTGACAATTT *Nde*I	
hb1	AAGCTTTCAGATTGCCCTGGTAG *EcoR*I	618 bp
hb2	TCTAGAGGGACACCCAGCTCA *Hind*III	

### Construction of the complemented *fur* strain

The complementation sequence of the *fur* gene (618 bp, including the 411-bp *fur* gene and its promoter region) in *Xv*17 was amplified using primers hb1 and hb2 (Table 2). The DNA fragment was cloned into pML123 to generate pMLfur (Table 1), which was transferred into the mutant strain fur-m by triparental conjugation. One transconjugant named fur-c was identified through screening on YDC (amended with Rif, Km and Gm (Table 1)). All obtained plasmids and *Xv* strains were confirmed by PCR and DNA sequencing.

### Detection of siderophore production

Siderophore production was measured using chrome azural S (CAS) agar plates and solutions [19]. Bacterial cell suspensions (OD_600_ = 1.0) were spotted onto a CAS plate and incubated at 28°C for 2 days. Siderophore production was indicated by the presence of a yellow halo around the bacterial colony. To measure siderophore production quantitatively, *Xv*17, fur-m and fur-c were cultured in LB medium at 28°C, and 0.5 ml of cell suspension of each *Xv* strain was mixed with 0.5 ml of CAS assay solution every 3 h for 36 h. Two hours after each mixing, the absorbance at 630 nm was measured using a spectrophotometer (Thermo Fisher Scientific). Siderophore production was calculated as follows: [(Ar-As)/Ar]×100%, where Ar and As represent the absorbance of uninoculated and inoculated media, respectively. There were three replicates per treatment and the experiment was repeated three times.

### Analyses of biofilm formation

Overnight cultures of *Xv*17, fur-m and fur-c were adjusted to an OD_600_ of 1.5. One hundred microliters of each cell suspension was transferred into 10 ml of LB broth in glass test tubes. The liquid cultures were incubated at 28°C for 5 days, and then, the broth was poured out slowly. After drying at 37°C for 1 h, biofilms on the surface of the test tubes were stained with 0.1% methyl violet for 30 min. A ring of violet precipitate developing on the inner wall of the tube indicated biofilm formation. Biofilm formation was also analyzed quantitatively by solubilizing the stained biofilms with 95% ethanol for 1 h and measuring the OD_590_ of the stained suspension with a spectrophotometer [20].

### Detection of quorum sensing (QS) signaling molecules

To assay for the production of QS signaling molecules, *A*. *tumefaciens* strain NTL4 (pZLR4) was cultured in 10 ml of AB minimal medium (ABM) [21] at 28°C for 12 h. Six milliliters of the overnight culture and 100 μl of X-gal (20 mg·ml^-1^) were added to 100 ml of ABM plates (1.2% agar). Two microliters of overnight cultures of strains *E*. *carotovora* subsp. *carotovora* Ecc-3, *Xv*17, fur-m or fur-c were spotted on each of the ABM plates separately and incubated at 28°C for 12–18 h. A blue halo around colonies indicated the production of QS signal molecules.

### Assay for swimming motility

The swimming motility was assayed as described previously [22] with some modifications. Overnight cultures of the *Xv* strains were adjusted to an OD_600_ of 0.5 with fresh LB medium. The swimming motility assay was initiated by spotting 2 μl of each cell suspension at the center of 0.3% LB agar plates amended with appropriate antibiotics. The plates were incubated at 28°C, and the halos formed by migrating bacteria were measured after 2 d. The colony diameter of each strain was also measured.

### Measurement of extracellular polysaccharide (EPS) production

EPS production was measured as described by Tang et al. [23] with some modifications. Cultures of each *Xv* strain were grown in LB medium for 4 d until their OD_600_ reached 2.5. After centrifuging at 10,000 rpm for 10 min, the supernatant was precipitated with ethanol and EPS was collected, dried at 37°C for 3 h and weighed.

### Virulence assay on tomato

Overnight cultures of *Xv*17, fur-m and fur-c were adjusted with fresh LB to an OD_600_ of 0.5. Thirty milliliters of each inoculum was spray inoculated to six 50-day-old tomato plants of a highly susceptible cultivar (Zhongshu No.4) under greenhouse conditions (25–30°C and 80% relative humidity). The number of leaf spots on the most severely infected leaf of each inoculated plant was counted to compare bacterial spot severity. LB medium was used as a negative control.

### Statistical analysis

All experiments in this study were performed at least three times. All values are shown as the mean ± standard deviation (SD). Data were subjected to ANOVA and Duncan’s multiple range tests using the Statistical Package for the Social Sciences (SPSS) software version 17.0. Differences were considered statistically significant at P<0.05.

## Results

### Confirmation of the *Xv fur* mutant and complementation strains

The *fur* gene encodes the Fur protein which is 136 amino acids in length based on the genome sequence of *Xv* [11] (GenBank accession number AF146022). PCR amplification of the *fur* mutant strain fur-m with the fur1 and fur2 primers and the subsequent sequencing of the PCR product confirmed that strain fur-m contained a band of 2,218 bp, consisting of a truncated *fur* gene interrupted by the Gm cassette (data not shown). The presence of the Gm cassette (855 bp) in fur-m was further confirmed by Southern blot and was absent from the wild-type strain *Xv*17 (data not shown). The fur-m strain was stable after continuous culturing for 20 generations in YDC medium. The fact that the fur complementation strain fur-c was Km^R^ suggested the successful transfer of the plasmid pMLfur into the fur-m strain. The presence of pMLfur in fur-c was further confirmed by PCR using the hd1 and hd2 primers, as two PCR bands were amplified as expected. One band was 618-bp, amplified from pMLfur DNA, and the other was 1,264-bp, amplified from fur-m DNA (data not shown).

### Role of *fur* in *Xv* siderophore production

**Fig 1:**
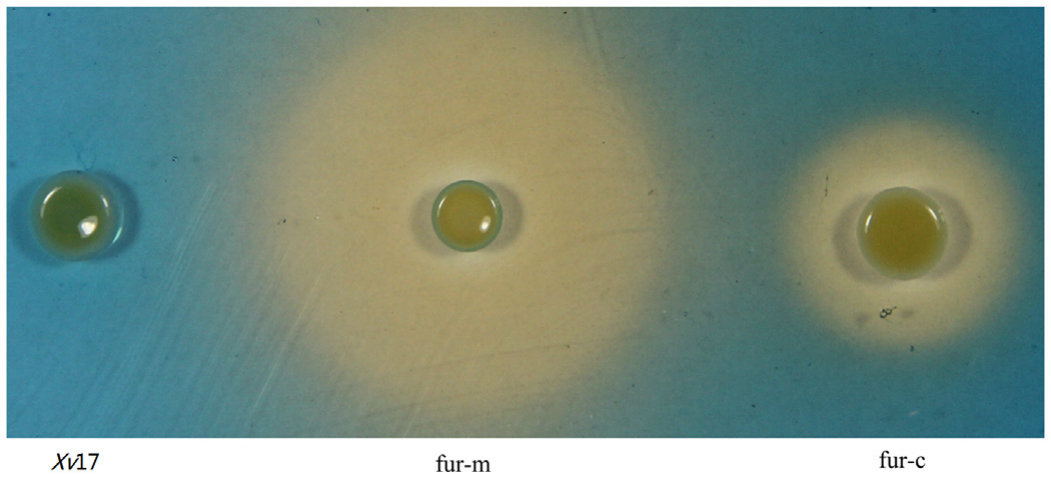
Comparison of siderophore production in *Xv* strains using the Chrome azurol S assay. Yellow halos around bacterial colonies indicate siderophore production. *Xv*17: wild-type *Xv* strain; fur-m: *fur* mutation strain of *Xv* 17; and fur-c: *fur* complementation strain of fur-m.

**Fig 2:**
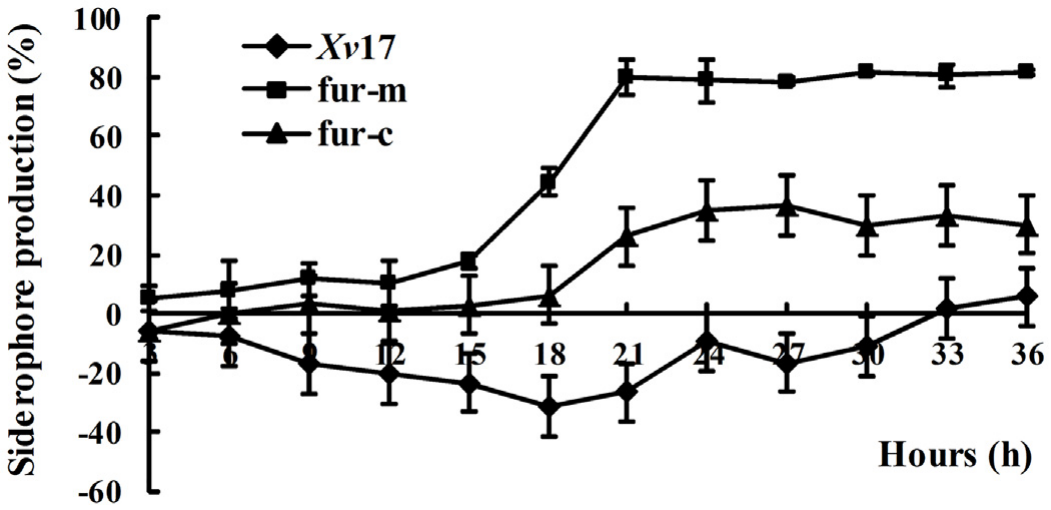
Quantitative comparison of siderophore production in *Xv* strains. Black diamond: wild-type strain *Xv*17; black square: *fur* mutation strain fur-m; and black triangle: *fur* complementation strain fur-c.

The *fur* mutant strain fur-m and the *fur* complemented strain fur-c showed increased siderophore production relative to the wild-type strain *Xv*17 as indicated by the presence of yellow halos around their colonies (Fig 1). *Xv*17 lacked a yellow halo, indicating undetectable levels of siderophore production (Fig 1). These results suggested that the increased siderophore production phenotype may be due to the absence of the *fur* gene. Siderophore production for fur-m and fur-c were slight at 15 h and increased to a maximum level at 21 and 27 h after incubation, respectively (Fig 2). From those times on, the siderophore production remained fairly constant. Maximum siderophore production by fur-m and fur-c was 81.21% at 21 h and 36.44% at 27 h, respectively (Fig 2). However, *Xv*17 produced little siderophore. Siderophore production in fur-m was significantly higher than that in fur-c throughout the experiment (P<0.05). However, the siderophore production phenotype of fur-m was not completely complemented by an expression vector containing the wild-type *fur* gene (Fig 2).

### Role of *fur* in *Xv* biofilm formation

**Fig 3:**
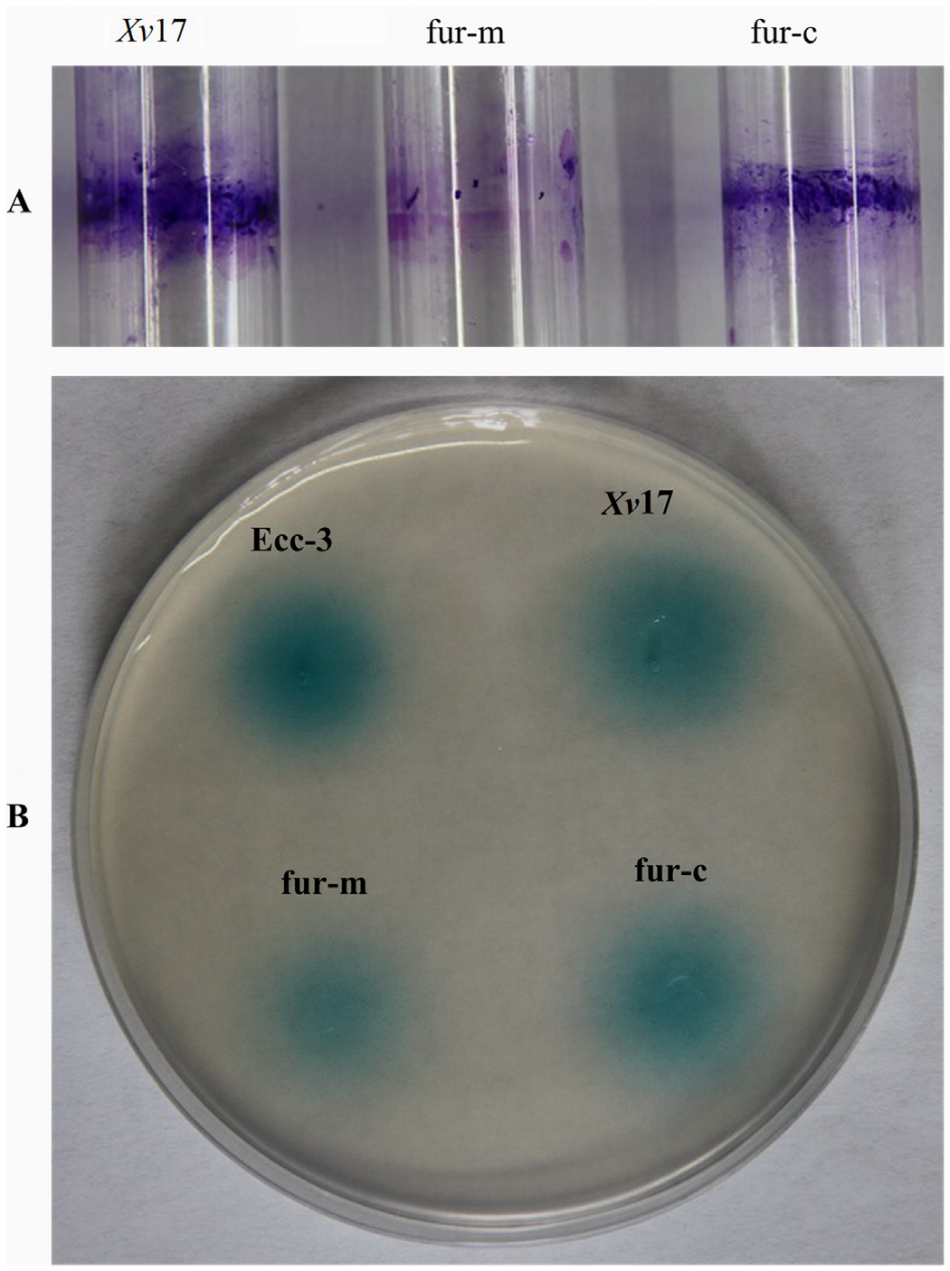
Effect of *fur* on biofilm formation (A) and quorum sensing (QS) signals in *Xv* (B). Biofilm formation is indicated by the ring formed on the surface of glass test tubes. QS signal expression was indicated by blue halos 12–18 h after each strain was spotted and after incubation on ABM plates at 28°C. A QS assay was used to detect signaling molecules as a blue halo. *Xv*17: wild-type *Xv* strain; fur-m: *fur* mutation strain of *Xv*17; and fur-c: *fur* complementation strain of fur-m. Ecc3: *E*. *carotovora* subsp. *carotovora* strain 3 as a positive control for expression of QS signals.

Because biofilm formation may influence the *Xv* colonization of tomato seed (data not shown), we investigated whether *fur* plays a role in biofilm formation by *Xv*. Our qualitative biofilm assay revealed that *Xv*17 and fur-c formed a ring of biofilm on the surface of glass test tubes, whereas fur-m did not under our conditions (Fig 3A). This observation was confirmed by our quantitative biofilm assay, as the mean absorption value of the biofilm by fur-m (0.058) was significantly lower than that of *Xv*17 (0.14) and fur-c (0.15) (P<0.05) (Table 3).

**Table 3:** Quantitative measurement of biofilm formation as indicated by absorbance at OD_590_ in *Xv*.

Strain	Absorbance value at OD_590_
*Xv*17	0.1395±0.0180 ^a^*
fur-m	0.0576±0.0082 ^b^
fur-c	0.1502 ±0.0220 ^a^

*Values are means of three Experiments with three replicates in each experiment. Values followed by different letters are significantly different (*P* < 0.05) based on Duncan’s multiple range test.

### Involvement of *fur* in QS

To determine whether the *fur* gene was involved in *Xv* QS, we compared *Xv*17, fur-m and fur-c in terms of their involvement in QS signaling molecule production (Fig 3B). *Xv*17 and fur-c produced a blue halo that was similar to that of Ecc-3 (positive control), indicating QS signal molecule production, whereas fur-m had a smaller and lighter blue halo (Fig 3B).

### Role of *fur* in swimming motility

**Fig 4:**
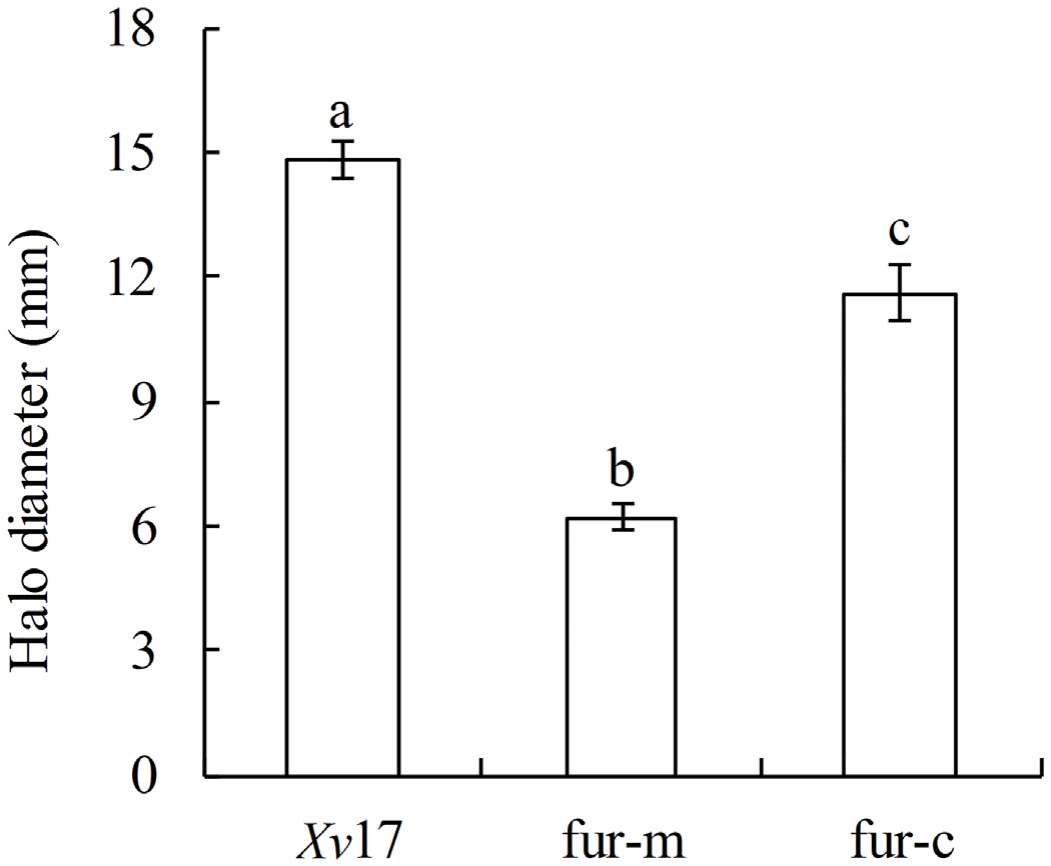
Comparison of swimming motility in *Xv* strains. Bar heights are the mean diameter of the swimming halos from three replicates ± standard deviation (SD). Different letters above the bars indicate significantly different diameters (*P* < 0.05) based on Duncan’s multiple range test.

Our assays for swimming motility revealed that halos formed by *Xv*17 and fur-c expanded to more than 11 mm in two days, whereas those by fur-m did not expand to more than 7 mm. fur-c largely restored the swimming ability of fur-m, but its halo diameter was still significantly smaller than that of *Xv*17 (*P <* 0.05). *Xv*17 (14.83 ± 0.45 mm), fur-m (6.20 ± 0.30 mm) and fur-c (11.60 ± 0.69 mm) differed significantly in their swimming abilities based on the diameters of their halos (*P <* 0.05) (Fig 4).

### Involvement of fur in *Xv* EPS production

**Fig 5:**
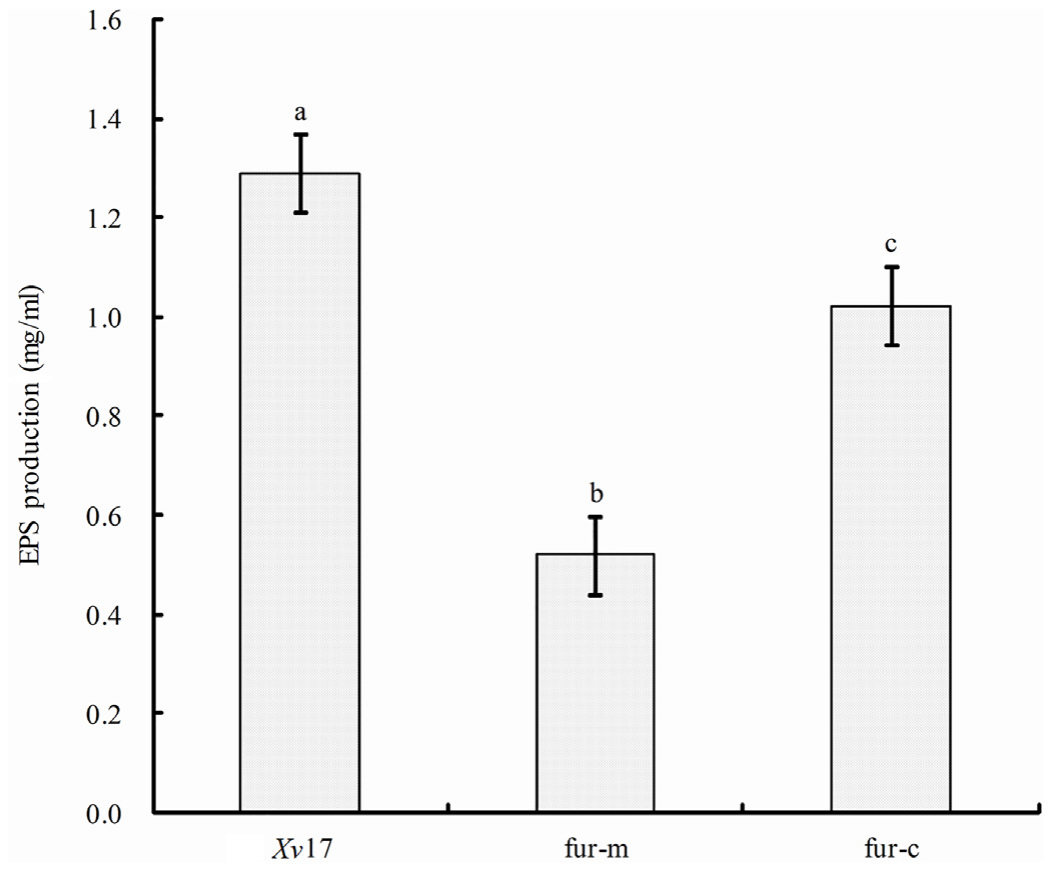
Effect of *fur* in extracellular EPS production in *Xv*. *Xv*17, fur-m and fur-c were cultured, and their EPS was dried and weighed. Bar heights are the mean EPS weights from three experiments with three replicates in each experiment. Different letters above the bars indicate significantly different EPS weights (*P* < 0.05) based on Duncan’s multiple range test.

After 4 days of growth in liquid LB medium, fur-m produced 0.52 mg of EPS precipitate per ml of culture compared to 1.28 mg for *Xv*17 and 1.02 mg for fur-c (Fig 5). fur-m produced significantly less EPS than *Xv*17 and fur-c (*P* < 0.05) (Fig 5).

### Contributions of *fur* to *Xv* virulence

**Fig 6:**
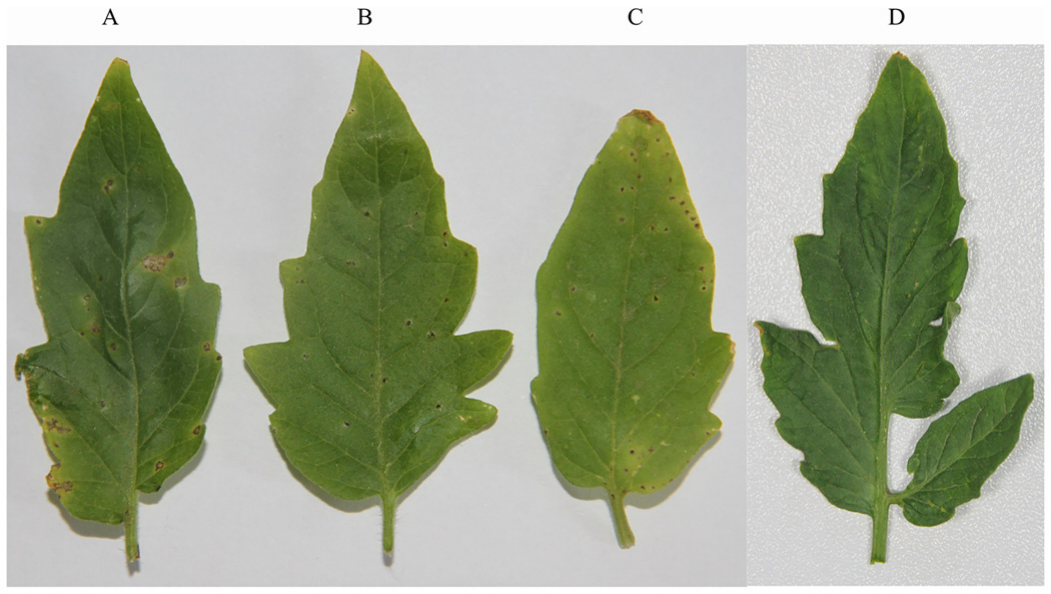
Effect of *fur* in *Xv* virulence on tomato leaves. Fifty-day-old tomato plants (cv No.4 Zhongshu) were spray inoculated with A) *Xv*17; B) fur-m; C) fur-c; and D) LB medium (negative control).

The bacterial spot severity caused by the mutant strain fur-m in tomato leaves was less than that of the wild-type strain *Xv*17. *Xv*17 produced blackish-brown spots surrounded by a yellow halo (Fig 6A), and its mean number of leaf spots was 10.33 ± 1.03 (Table 4). In contrast, only black spots appeared on the leaves that were spray-inoculated with the mutant strain fur-m. Moreover, the spots were smaller and did not develop a yellow halo (Fig 6B). The spots of *Xv*17and fur-c appear to be similar (Fig 6C). fur-m produced fewer spots per leaf (4.67 ± 0.81) than *X*v17. The number of spots elicited by the complementation strain fur-c (10.50 ± 1.05) was not significantly different from that of *Xv*17 (Table 4) (*P* < 0.05). The difference in virulence between *Xv*17 and fur-m was significant (*P* < 0.05).

**Table 4:** Virulence of *Xv* strains on tomato leaves.

Strain	Mean # of leaf spot^x^
*Xv*17	10.33 ± 1.03 ^a^
fur-m	4.67 ± 0.70 ^b^
fur-c	10.50 ± 1.05 ^a^

^x^Virulence was tested on 50-day-old tomato plants (cv No. 4 Zhongshu) that were spray-inoculated with each tested strain separately. Values are the mean numbers of leaf spots per leaf (n = 6). Values followed by different letters are significantly different (*P* < 0.05) based on Duncan’s multiple range test.

## Discussion

Although Fur has been studied in a number of plant pathogenic bacteria including *X*. *oryzae* pv. *oryzae*, *X*. *campestris* pv. *campestris* and *Edwardsiella tarda* [5–8], its role in *Xv* has not been previously studied. We investigated the functions of fur in *Xv* by constructing a *fur* mutant strain fur-m. Compared with wild-type strain *Xv*17 which did not produce any siderophore, fur-m produced a significant amount of siderophore, indicating that *fur* plays an important role in the negative regulation of siderophore synthesis in *Xv*. In addition, biofilm formation, the production of QS signaling chemicals, swimming motility, EPS production and virulence on tomato were all significantly reduced in fur-m, suggesting that *fur* in *Xv* plays an important role, either directly or indirectly, in the regulation of these virulence or virulence-related functions.

Similar to other bacteria, such as *X*. *campestris* [24], *Pseudomonas aeruginosa* [25], and *Bacillus subtilis* [26], Fur negatively regulates siderophore synthesis in *Xv*, as evidenced by the presence of yellow halos around the colonies of mutant strain fur-m but the absence of such halos around wild-type strain *Xv*17. In addition, when the *fur* mutation was complemented by a wild-type *fur* gene in a complementation vector (pMLfur), the resulting strain fur-c significantly reduced the siderophore production compared to fur-m, further suggesting that *fur* is involved in the negative regulation of siderophore synthesis in *Xv*. Why fur-c still produced a significant amount of siderophore 15–36 h after growth remains unclear (Fig 2), as the fur complemented strains in *X*. *campestris* did not produce any visible amount of siderophore [24]. The *fur* gene may need to be present in cis in *Xv* genome, not provided in trans in a plasmid, to completely inhibit siderophore synthesis and fully restore the production of EPS and swimming ability in *Xv*.

Bacterial biofilms are considered to be resistant to environmental stress on the plant surface [27–29] and to offer protection from the antimicrobial compounds secreted by plants [27,30]. When *fur* was mutated, the mutant strain produced little biofilm relative to the wild-type and the fur complementation strains, suggesting that *fur* is important for biofilm formation in *Xv*.

Quorum sensing regulates a variety of physiological functions in bacteria, including motility, conjugation, competence, sporulation, secretion, antibiotic production, virulence and biofilm formation [29, 31]. Numerous *Xanthomonas* species have evolved QS systems for genetic regulation at the community level [32]. *fur* has been reported to control the expression of the *psy*R and *psy*I genes involved in QS in *Pseudomonas syringae* pv. *tabaci* [2]. Previous studies have demonstrated that N-acyl homoserine lactone (N-AHL) [33], *LuxR* and *LuxI* [34], *las*I/*rhl* I [35], *qscR* [36] and *aiiA* [37] are all involved in QS and contribute to virulence on their host plants. Our results indicated the reduced production of QS signal molecules in fur-m, suggesting either a direct or indirect relationship between *fur* and QS.

Motility is considered to be an important epiphytic fitness trait, enabling bacterial cells to locate resources and access sites that allow them to avoid environmental stresses [38]. Motility regulation is associated with N-AHL-dependent genes (QS system) in *P*. *syringae* [33] and the GacS/GacA system in *P*. *aeruginosa* [39]. GacS/GacA-dependent gene regulation can be considered as part of the QS machinery [40]. Our results suggest that *fur* may be at least partially involved in regulating *Xv* motility because the swimming motility was significantly reduced for the *Xv fur* mutant strain. Although *fur* was related to QS and swimming, it was not clear whether swimming motility was regulated by the QS and Gac systems in *Xv*. Moreover, bacterial motility is a critical virulence factor because it facilitates pathogen entry into plant tissues [41]. EPS produced by pathogens are thought to be involved in adhesion, biofilm maintenance [42], heavy metal stress tolerance [43] and plant disease symptom development [44]. Many genes are known to regulate bacterial EPS production in vivo, including *zur* of *X*. *oryzae* pv. *oryzae* [45]; *hrp in Xv* [46]; the *che*, *flg*, *flh* and *fli* genes in *E*. *coli* K-12 strains [47]; and the *luxR* homologue in *Vibrio alginolyticus* [48]. Moreover, the deletion of these genes often results in the reduction of bacterial virulence. Deletion of the *Xv fur* gene significantly compromised EPS production. However, bioinformatic analysis indicates that the promoter regions of the above genes related to EPS production do not contain the Fur box to which Fur binds (data not shown). Thus, the mechanism by which Fur regulates EPS production remains the subject of future studies.

*Xv* is a serious threat to tomato and pepper production. *fur* has been found to be important for the virulence and pathogenicity of a number of pathogenic bacteria in animal and plant hosts [2,5,24,49,50]. The virulence of the *Xv fur* mutant strain was significantly reduced in our study, suggesting that *fur* contributes to *Xv* virulence on tomato. A previous study has shown that the rapid accumulation of reactive oxygen species in the plant is closely related to plant disease [51]. Bacteria could take advantage of the generation of active oxygen species to kill the host cells during invasion and colonization [52,53]. The *X*. *campestris fur* mutant was vulnerable to oxidative stress, which may at least partially account for the attenuated virulence phenotype of the *fur* mutant [54]. It is not surprising that our *fur* mutant was reduced in virulence, as in our study, many of the virulence or virulence-related factors, such as QS, biofilm formation, motility and EPS production, were all significantly reduced when *fur* was mutated. Our results suggest that in addition to siderophore production, *fur* also contributes significantly to other important physiological functions that directly or indirectly lead to the virulence of *Xv* in its host plants. Future research is needed to determine whether QS and the virulence factors, such as EPS production, biofilm formation and motility, are regulated directly by Fur or indirectly due to increased iron levels in fur mutant strains.
